# Iron-Based Superconducting Nanowires: Electric Transport and Voltage-Noise Properties

**DOI:** 10.3390/nano10050862

**Published:** 2020-04-30

**Authors:** Sergio Pagano, Nadia Martucciello, Emanuele Enrico, Eugenio Monticone, Kazumasa Iida, Carlo Barone

**Affiliations:** 1 Dipartimento di Fisica “E.R. Caianiello”, Università degli Studi di Salerno, I-84084 Fisciano, Salerno, Italy; cbarone@unisa.it; 2 CNR-SPIN Salerno, c/o Università degli Studi di Salerno, I-84084 Fisciano, Salerno, Italy; nadia.martucciello@spin.cnr.it; 3 INFN Gruppo Collegato di Salerno, c/o Università degli Studi di Salerno, I-84084 Fisciano, Salerno, Italy; 4 Istituto Nazionale di Ricerca Metrologica, I-10135 Torino, Italy; e.enrico@inrim.it (E.E.); e.monticone@inrim.it (E.M.); 5 Department of Materials Physics, Nagoya University, Nagoya 464-8603, Japan; iida@mp.pse.nagoya-u.ac.jp

**Keywords:** iron-based superconductors, nanowires, single-photon detectors

## Abstract

The discovery of iron-based superconductors paved the way for advanced possible applications, mostly in high magnetic fields, but also in electronics. Among superconductive devices, nanowire detectors have raised a large interest in recent years, due to their ability to detect a single photon in the visible and infrared (IR) spectral region. Although not yet optimal for single-photon detection, iron-based superconducting nanowire detectors would bring clear advantages due to their high operating temperature, also possibly profiting of other peculiar material properties. However, there are several challenges yet to be overcome, regarding mainly: fabrication of ultra-thin films, appropriate passivation techniques, optimization of nano-patterning, and high-quality electrical contacts. Test nanowire structures, made by ultra-thin films of Co-doped BaFe_2_As_2_, have been fabricated and characterized in their transport and intrinsic noise properties. The results on the realized nanostructures show good properties in terms of material resistivity and critical current. Details on the fabrication and low temperature characterization of the realized nanodevices are presented, together with a study of possible degradation phenomena induced by ageing effects.

## 1. Introduction

Most widespread applications of superconducting nanowires regard their use as single-photon detectors, due to their ability in detecting single photons in the visible and IR spectral region [1,2]. Moreover, their peculiar physics has brought to the development of novel electronic cryodevices, such as pulse discriminators [3,4], logic gates [5], and also memory elements [6]. 

The interest in superconducting nanowires has also recently increased, due to their possible application in quantum technologies, including quantum sensing and computing. This has been highlighted in the case of YBa_2_Cu_3_O_7-x_ (YBCO) nanowires with phase-slip dynamics, where evidence of energy-level quantization in the nanowires has been reported [7]. Moreover, it has also been shown that the absorption of a single photon changes the quantum state of the nanowire, an important result for the development of single-photon detectors with high operating temperature and superior temporal resolution [7].

Although traditional superconducting nanowire single-photon detectors (SNSPDs) have the advantage of offering single-photon sensitivity, combined with low dark count rates [8], low jitter [9], short recovery times, and free-running operation [2], one drawback is their low operating temperature. This is essentially due to the fact that current SNSPDs are fabricated using mostly conventional low-temperature superconductors, such as NbN and WSi [10,11], in order to achieve high sensitivity and to simplify nanofabrication processes. Several efforts have been made to realize nanowires with high critical temperature (*T_c_*) materials, including MgB_2_ and cuprate superconductors [12,13,14,15]. The recently discovered iron-based superconductors have attracted great interest to explore their potentialities in the field of large-scale current transport [16,17] and in microelectronics or nanoelectronics applications [18,19]. These compounds could pave a new way to the fabrication of superconducting nanowires, also profiting of intrinsic material properties to improve detection performances (in particular, speed and efficiency). In this respect some preliminary results are reported in [20]. However, there are several issues to be taken into account that are mainly related to the occurrence of non-hysteretic current-voltage characteristics (no switching), to the difficulty in fabricating ultra-thin films (dead layer problem of high-*T_c_* compounds), and to the easy surface degradation.

In order to overcome these difficulties and address these challenges, we have realized and tested a number of nanowires made of iron-based superconductors. A detailed investigation of their electric transport properties is reported in this work. In addition, a study of the charge carrier fluctuation effects, in a wide temperature range, has been carried out, in order to get useful information on the physics of the nanowires for the optimization of nanopatterning processes and for the realization of high-quality electrical contacts. To our knowledge, there are no other noise measurements on iron-based nanowires reported in the scientific literature. By means of noise spectroscopy, moreover, a deeper understanding of physical mechanisms and of ageing-induced degradation effects can be obtained, as already demonstrated for graphene [21,22], for iron-chalcogenide [23], and for the same Co-doped BaFe_2_As_2_ superconductors [24] used here. The reported experimental results, together with theoretical interpretations, may be useful in view of the fabrication of more performant and usable nanodevices.

## 2. Materials and Methods

A necessary starting point to realize a nanowire detector is a very thin superconducting film, ideally with a thickness of the order of the material coherence length, in order to maintain the superconductive state and maximize the photon energy sensitivity. However, the growth mechanism of high-*T_c_* superconductors is complex and often results in a “dead” layer at the interface with the substrate. Therefore, a compromise between thickness and film quality has to be achieved. In this respect, Co-doped BaFe_2_As_2_ superconducting thin films, with a thickness of about 20 nm, were grown, by using a pulsed laser deposition (PLD) technique, on 0.5-mm-thick CaF_2_ (001) substrates. The choice of the Co doping level is done in order to optimize the superconducting transition temperature of the material [25]. This resulted in a nominal composition of the PLD target of Ba:Fe:Co:As = 1:1.84:0.16:2. The fabrication parameters, such as laser repetition frequency (7 Hz) and growth temperature (700 °C) [26], together with the choice of CaF_2_ substrate, were selected in order to optimize the *T_c_* of the films, as well as other material properties [27]. The high phase purity of the target material and of the samples was observed with X-ray diffraction (Malvern Panalytical Ltd., Malvern, UK) and by using transmission electron microscopy (JEOL Ltd., Tokyo, Japan) the absence of appreciable defects was also verified [26]. Moreover, to reduce possible degradation effects due to ageing, an in-situ passivation process was employed, by depositing a thin layer of magnesium aluminate (MgAl_2_O_4_) spinel (MAS). Its high resistance against chemical attack, high thermal shock resistance and compatibility with a large variety of metals are important properties that make MAS very advantageous as a protective cap layer [28].

**Figure 1:**
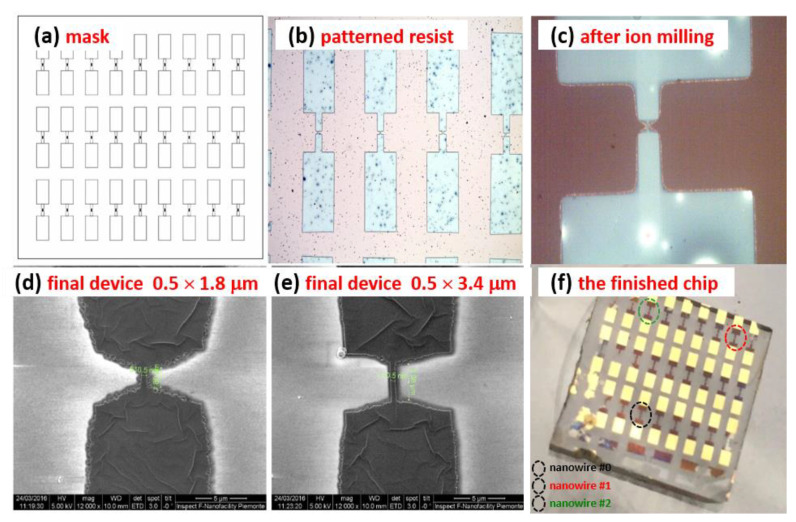
Microphotographs and images representing the different steps of the nanowires fabrication process: from the used mask design (**a**) to the finished 1 cm^2^ chip, after electronic and optical lithography (**b**–**f**). The devices, whose experimental results are here reported and discussed, are evidenced with colorized circles.

Subsequently, the superconducting films were patterned in the shape of several nanowires using a combination of optical and e-beam lithography and ion beam etching. The design used for the geometry definition, shown in Figure 1a, consists of a series of nanowires with a nominal width and length of 500 nm and 5 μm, respectively, each connected to 1 mm × 0.5 mm bonding pads. A 100 nm thick resist layer, type ma-N 2401, was used for reproducing the design on the samples (Figure 1b). 

A subsequent ion milling process (Figure 1c) defined the nanowire geometry. After etching, due to nanolithography process imperfections, the final devices realized showed different lengths, ranging from 1.8 (Figure 1d) to 3.4 μm (Figure 1e), while keeping the same width. Finally, to reduce the contact resistance, a thin layer of Ti/Au (5/50 nm) was deposited after Ar ion cleaning, on the contact pads area, obtaining the finished chip consisting of 27 separated devices. Among these, the ones here investigated are evidenced with circles of different colors and refer to pristine (black circle) and to two years-aged (red and green circles) nanostructures (see Figure 1f, for details).

**Figure 2:**
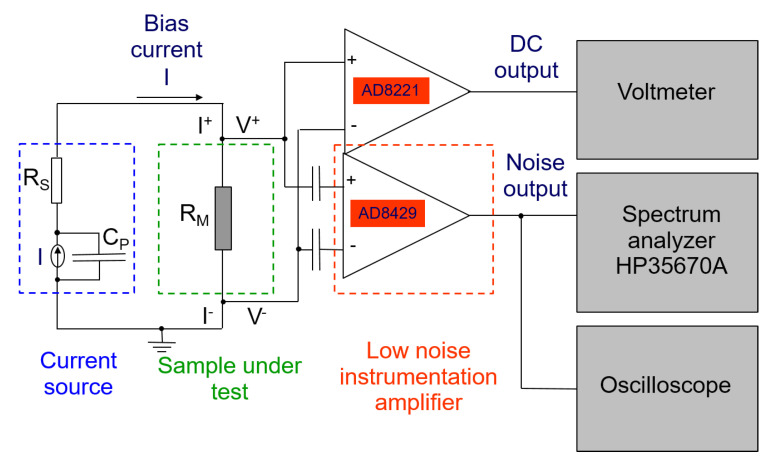
Schematic of the bias and readout electronics. The colored dashed boxes enclose the bias circuit (blue), the device under test (green), and the instrumentation amplifier (red). See text for details.

All the electrical characterizations were performed in a closed cryocooler system, characterized by an operation temperature range from 8 to 325 K, with a temperature stabilization better of 0.2 K. Low-noise DC bias, and DC and AC readout electronics were used to record the sample electrical response, as shown Figure 2 [29]. In particular, the measurements were performed by using a two-probe technique (green box of Figure 2). A low-pass passive filter was inserted at the input of the bias current source, with a user variable series resistance *R_S_*, much larger than the sample resistance *R_M_*, and a cutoff frequency of few Hz (blue box of Figure 2). The output AC voltage was amplified by a low-noise instrumentation amplifier, model AD8429 having 1 nV/$\sqrt{\mathrm{Hz}}$ noise level, (red box of Figure 2), and its spectral analysis was done by a dynamic signal analyzer model HP35670A. The DC voltage was amplified by a low noise instrumentation amplifier, model AD8221, and recorded by a digital voltmeter. The absence of unwanted contact noise contribution was verified by resorting to a specific procedure [30], whose validity has been already tested in the case of superconductors [31], innovative carbon nanotube and photovoltaic devices [32,33], and magnetic compounds [34,35]. The lowest noise level measured, corresponding to the instrumental background, was 1.71 × 10^−18^ V^2^/Hz.

## 3. Results

### 3.1. DC Electrical Transport Measurements

**Figure 3:**
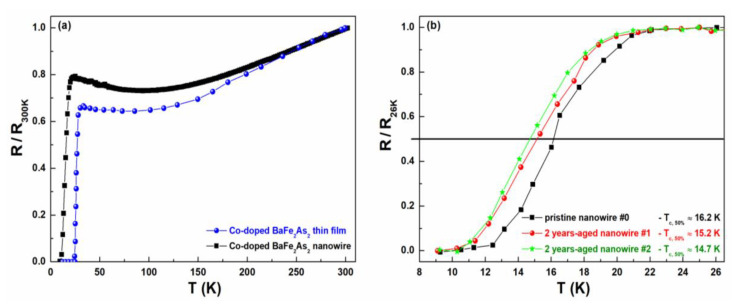
Resistance versus temperature plots. (**a**) The data refer to the resistance of thin films (blue circles) and of nanowires (black squares), normalized to their room temperature values. (**b**) An enlargement of the superconducting transition region is shown, where the resistances, normalized to their normal state values at *T* = 26 K, are reported for pristine and two years-aged nanostructures.

The temperature dependence of the resistance of Co-doped BaFe_2_As_2_ nanowires was very similar to what already found for thin films [27]. In particular, the data in Figure 3a show a metallic behavior for temperatures higher than 100 K, both for the thin film (blue circles) and for a nanowire (black squares). A minimum of the resistivity was observed around 100 K, with an upturn down to the onset of superconductivity ($T_{c}^{onset}$), which was about 30 K for the thin film and 24 K for the nanowire. The difference in critical temperature was due to different film thickness, much larger in the case of the thin film. The resistance increase, observed by decreasing the temperature from 100 K to $T_{c}^{onset}$, can be attributed to the occurrence of localization effects [36]. In this respect, a detailed analysis, reported in [37], limits to variable-range hopping (VRH) or to weak-localization (WL) effects the possible explanation of the resistivity behavior. The DC measurements alone were not able to distinguish between the two mechanisms and, therefore, additional experimental investigations, such as noise spectroscopy, are necessary. 

The results obtained by studying the fluctuation processes are shown in the following and will be useful to clarify this issue. The most evident difference between thin films and nanowires concerns the value of *T_c_*. More in details, for the single nanowire the superconducting transition, defined at 50% of the normal-state resistance *R_N_*, occurred at around 16 K and was significantly lower than that of the thin film. This fact, as already discussed above, was due to the choice of fabricating a very thin film for the nanowire sample, in order to boost the device sensitivity. The observed low *T_c_* was, however, larger than that of standard NbN nanowires (about 11 K). Moreover, as shown in Figure 3b, where the normalized resistance was reported in the superconducting transition region, after two years of ageing the nanodevices maintained comparable critical temperatures. This finding gives the important indication that iron-based nanowires, realized with the passivation process described above, do not show degradation of their superconducting properties after up to two years of storage at room temperature and normal atmosphere. This result might be very useful in technological applications requiring long-time operation and stable performances, as also strengthened by an evident homogeneous distribution of the critical temperatures all over the chip area. Such a feature is confirmed by the experimental data of Figure 3b, which refer to differently positioned nanowires.

**Figure 4:**
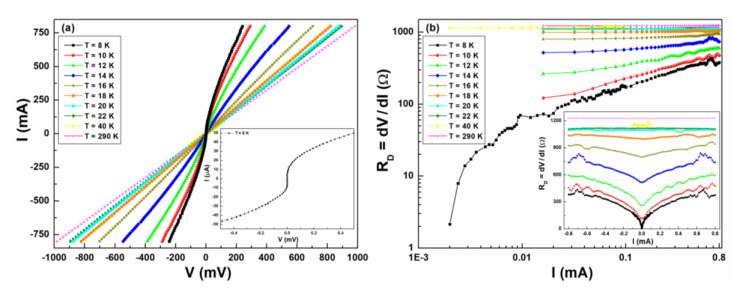
Current–voltage (*I–V*) characteristics. (**a**) The different curves refer to temperatures from 8 to 290 K. An enlargement of the *I–V* curve at the temperature of 8 K is shown in the inset. (**b**) The DC current dependence of the differential resistance *R_D_* is reported at different temperatures in the log-scale. In the inset, the full current range, spanning from negative to positive values, is shown using a linear-scale.

All the realized nanowires have non-hysteretic current–voltage (*I–V*) curves down to a temperature of 8 K, as shown in Figure 4a. Although the absence of degradation is a positive feature, the absence of hysteresis in *I–V* curves in the superconducting state is, instead, a negative point, when aiming at developing SNSPDs. It cannot be excluded that, by further lowering the temperature, a hysteretic *I–V* curve could be observed. However, as our aim was to realize high operating temperature nanowires, this is not of interest for this work. A critical current *I_c_* of the order of 10 μA is measured at 8 K, as shown in the inset of Figure 4a. A non-hysteretic behavior was also reported in [20] for iron-based superconductors and in [38,39] in the case of YBCO. Some hysteresis was observed for a YBCO microbridge (not a nanowire) at *T* = 4 K [40]. Compared to the reported results, the nanowires presented here have, however, the advantage of showing higher resistance values, similar to that of the low-*T_c_* nanowires. Figure 4b shows the DC current dependence of the nanowire differential resistance *R_D_* for various temperatures. A gradual change of *R_D_* can be observed in the temperature range across the superconducting transition up to the normal resistance value, of about 1200 Ω. In the inset are clearly visible abrupt changes in *R_D_* occurring at bias current of about 0.6–0.7 mA, both for positive and negative values. These are most probably due to the transition to the normal state of the large area structures (see Figure 1b). Overall, it is evident that iron-based nanowires do not yet show characteristics optimal for light detection. In order to get more information on the transport processes, noise measurement have been employed.

### 3.2. Voltage–Noise Spectral Density Measurements

The basic properties of random data in the time domain, known as noise or fluctuations, are described by the autocorrelation function. In the frequency domain, information on the noise mechanisms in action are obtained by the spectral density function *S*_x_, which is the Fourier transform of the autocorrelation function, as derived from the Wiener–Khintchine theorem [41]. In condensed matter physics, the noise quantity usually measured is the spectral density of voltage fluctuations, whose frequency composition *S_V_(f)* gives indications on the different electric noise contributions. In particular, the most common types of low-frequency noises are: (I) Johnson or thermal noise, that is generated by the thermal agitation of the charge carriers inside an electrical conductor at equilibrium and is defined as *S_V_ = 4k_B_TR* (with *k_B_* the Boltzmann constant, *T* the temperature, and *R* the electrical resistance); (II) the shot noise, that is originated from the discrete nature of the electric charge and is defined as *S_V_ = 2eIR^2^* (with *e* the electron charge, and *I* the bias current); and (III) the 1/*f* or flicker noise, that is related to the ensemble average of thermally activated two levels random resistance fluctuations, due to intrinsic material aspects, such as grain boundaries, defects, etc. [41]. The noise of type (I) and (II) has frequency-independent spectral density amplitude (“white noise”), while noise type (III) has a clear frequency dependence (“colored noise”). 

**Figure 5:**
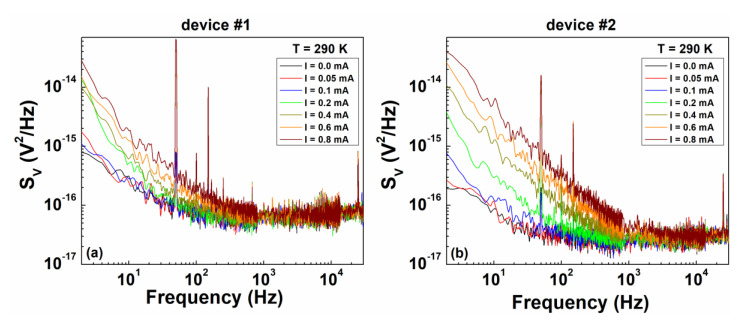
Voltage-spectral density traces. The frequency dependence of *S_V_* is shown for two different devices of the same chip, device #1 (**a**) and device #2 (**b**), at room temperature (290 K) and for bias currents ranging from 0 to 0.8 mA.

Both white and 1/*f* noise components were evident in the frequency dependence of *S_V_* for the iron-based nanowires investigated here, as shown in Figure 5 for two devices (nanowire #1 and nanowire #2 indicated in Figure 1f) and at the temperature of 290 K. More in details, the overall experimental noise spectral density can be modeled as:

(1) $$S_{V}\left(I,T\right)=\frac{K\left(I,T\right)}{f}+S_{0}+S_{B}$$

where *K(I,T)* is the 1/*f* noise amplitude, dependent on bias current and temperature; *S*_0_ is the “white noise” amplitude, which also may be bias and temperature dependent; *S_B_* is the instrumental background noise amplitude, in our case estimated to be a constant value of 1.71 × 10^−18^ V^2^/Hz (see Section 2). All the physical information on the transport processes resides in *K* and *S*_0_, whose analysis in temperature and in bias current can be very useful for understanding the dynamic processes of the charge carriers. It is worth noting that a similar approach has been used to study nanowires made of granular aluminum oxide [42].

**Figure 6:**
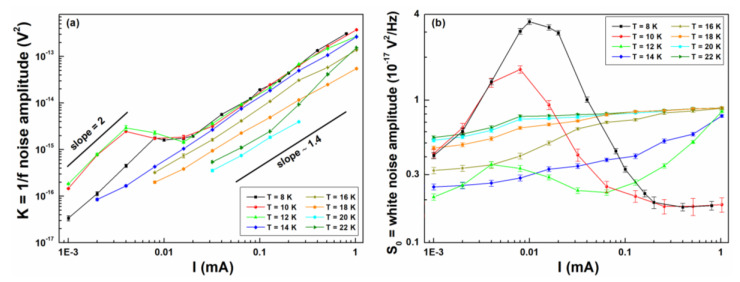
Electric noise amplitudes behavior. The dependencies on bias current, and at several temperatures, are shown for the 1/*f* (**a**) and for the “white” (**b**) noise components, respectively.

The experimental data can be interpreted in terms of the model described in Equation (1) to obtain the temperature and bias current dependence of *K* and *S*_0_. In Figure 6a,b are shown the DC current dependencies of the 1/*f* and “white noise” amplitudes, respectively, at various temperatures near *T_c_*. Figure 6a shows a noise peak occurring at both low temperatures and DC currents, with a characteristic slope of two, and a noise growth occurring at high temperatures and currents, with a characteristic slope of 1.4. Figure 6b shows a large noise peak at low currents and temperatures, in correspondence of the superconducting transition of the nanowire, and a noise increase at larger temperatures, almost current independent.

**Figure 7:**
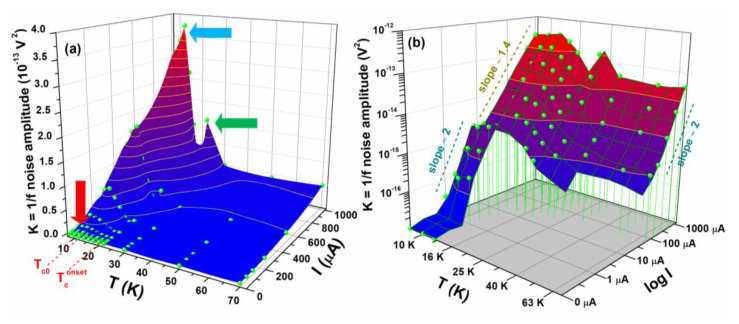
Amplitude of the 1/*f* noise component. The temperature and bias current dependencies of the 1/*f* noise are shown by using linear-axes scales (**a**) and logarithmic-axes scales (**b**) for the noise amplitude and for the bias current. The 1/*f* noise peaks at different temperatures are indicated with colorized arrows in panel (**a**). The bias current slopes are explicitly reported in panel (**b**).

To better appreciate the dependence of the noise on temperature and bias current, the experimental data can be shown in three-dimensional (3D) plots. In Figure 7a the 1/*f* noise amplitude *K* is plotted, with a linear temperature and bias current scale, and three noise peaks, evidenced by arrows, are clearly visible. The peaks indicated by the green and red arrows are expected and are due to percolation fluctuations occurring in the transition region of the nanowire (red arrow) and of the large area region (green arrow), respectively, where the superconductor is a mixture of normal metallic and superconducting phases [41,43,44]. On the contrary, the strong increase of the 1/*f* noise at low temperatures and high currents (blue arrow in Figure 7a) cannot be explained by reported theoretical interpretations and deserves more investigation in the next future. 

The same behavior of the 1/*f* noise amplitude is shown in Figure 7b by using a linear temperature x-scale and a logarithmic bias current and noise scale. This choice allows evidencing the power law dependence of the noise amplitude on bias current. The figure shows a (standard) quadratic dependence at low temperatures and below *I_c_*. This is expected according to a simple percolation model describing the full superconducting regime [41,43,44]. Unexpectedly, a current slope of 1.4 is observed for temperatures up to $T_{c}^{onset}$ and for higher currents than *I_c_*. This unusual finding may be connected with the unusual 1/*f* noise peak and could reveal the activation of additional noise sources that occurs in a region where the nanowire is already in the normal state but the thin-film part is still superconducting. This “intermediate” regime is characterized by a strong non-uniform conductivity, which could be responsible for both the high noise level and its non-standard bias current dependence. Additionally, the graph of Figure 7b shows that, at temperatures above $T_{c}^{onset}$, where the nanowire normal resistivity is characterized by a metal-insulator behavior, the current slope of the 1/*f* noise is again quadratic. This finding excludes the presence of WL effects, which appear as a linear current dependence of the 1/*f* noise component [45,46], while indicates the occurrence of VRH conductivity in the insulating regime and of resistance fluctuation processes in the metallic region [37,41].

**Figure 8:**
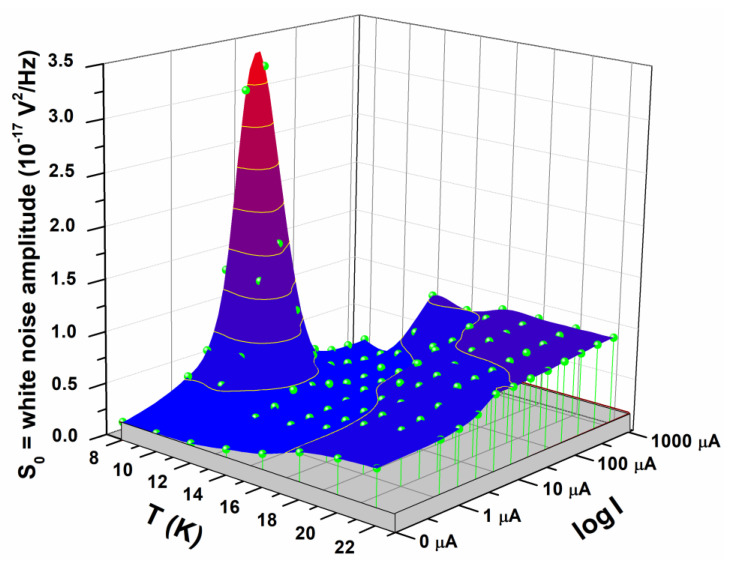
Amplitude of the “white noise” component. The temperature and bias current dependencies of the “white noise” are shown by using linear-axes scales for the noise amplitude and for the temperature, and logarithmic-axis scale for the bias current. The instrumental background noise is also shown as a gray floor.

The temperature and bias current dependence of the “white noise” *S*_0_ amplitude, shown in Figure 8, has a peculiar behavior. *S*_0_ has a maximum at low currents (centered near *I_c_*) and low temperatures (when the nanowire is superconducting), and seems to peak when the bias current reaches the critical one of the nanowire. Moreover, *S*_0_ has a smaller peak near $T_{c}^{onset}$, where the large area regions start their superconducting transition. At larger current and higher temperature, the “white noise” amplitude tends to become current independent and to approach the standard Johnson noise value. These experimental findings are new and, to our knowledge, not been reported in literature.

## 4. Conclusions

Nanowire structures were realized using ultra-thin films of Co-doped BaFe_2_As_2_. The sample fabrication and nanolithography techniques were optimized to preserve the superconducting properties of the material. The resulting critical temperature, about 16 K, was reduced of only 1 K after two years of room temperature and ambient atmosphere storage, showing that degradation effects due to ageing were not relevant. Due to their high superconducting transition temperature, iron-based nanowires represent an interesting alternative to NbN-based nanowires, whose critical temperature was about 10 K in ultra-thin film devices, for application as single-photon detectors. However, additional improvements in the fabrication technology are needed in order to achieve hysteretic *I–V* characteristic, a necessary ingredient for SSPD device. In this respect, a deep investigation of the transport mechanism in these devices is important, and it has been shown that noise spectroscopy can provide useful information. The study of the noise sources and their effect on dark counts, combined with the photo-response analysis, is currently in progress with the aim of developing useful single photon detectors operating at temperature above 10 K.
